# Supplementary material for: Glycine soja Leaf and Stem Extract Ameliorates Atopic Dermatitis-like Skin Inflammation by Inhibiting JAK/STAT Signaling
Source: Int J Mol Sci. 2025 May 9;26(10):4560. doi: 10.3390/ijms26104560 (PMC12110808; doi:10.3390/ijms26104560)

Supplementary Materials

Supplementary method S1. *Induction of AD and application of GS extract in NC/Nga mice*

The animal experiment was approved by the Animal Experimentation Ethics Committee of the Korea Institute of Oriental Medicine (ethical approval code 22-078). Seven-week-old male NC/Nga mice (Central Laboratory Animal Inc., Korea) were randomly classified (n=6); (1) No treatment (normal), (2) DfE treatment (control), (3) DfE+GS extract 30 mg/kg (GS 30 mg/kg), (4) DfE+GS extract 100 mg/kg (GS 100 mg/kg), (5) DfE+GS extract 200 mg/kg (GS 200 mg/kg) group, or (6) DfE+Dexamethasone 3 mg/kg (Dex). The AD model was induced by DfE application as presented in Fig. 1. The mice were depilated the hair of back skin with a depilator. After 24 hours, 150 ul 4% SDS was applied to the back of each mouse. After 1 hour, except for the normal mice, Biostir AD ointment (Hyogo, Japan) was applied to the back and ear of each mouse (100 mg/mouse), twice a week for 3 weeks. GS extracts and dexamethasone were orally administered daily for 17 days.

Supplementary method S2. *Analytical conditions*

The Waters Aquity UPLC system equipped with a quaternary pump, auto-sampler, photodiode array detector with Acquity UPLC® BEH C18, 100 × 2.1 mm, 1.7 μm was used for analysis (Waters, MA, USA). An elution with solvent A (water) and solvent B (acetonitrile) in a gradient elution at a flow rate of 0.5 mL/min was carried out as followings: 0 - 2 min, 5-5% B; 2 – 5 min, 5 – 15% B; 5 – 30 min, 15 – 55% B; 30 – 35 min, 55 – 72% B; 35 – 40 min, 72 - 90% B; 40 – 42 min, 90 – 100%B; 42 – 44 min, 100 – 5% B; 44 – 45 min, 5 – 5% B. The column temperature was kept at 40ºC and the injection volume was 2 µL. The Qda equipped with an ESI (electrospray ionization) source and MS was used to obtain the mass spectra. The analysis of ESI source was on negative and positive ion. The optimum ESI source parameters were acquired at Capillary 8kV and Probe 600℃. Nitrogen gas was used as curtain, collision, nebulizer, and heating gas. Acetonitrile, Ethanol, and methanol were obtained from J. T. Baker (USA).

Supplementary method S3. *Quantitative reverse transcription polymerase chain reaction* (*RT-qPCR)*

Total RNA from the mouse skin tissue and collected cells was extracted using a HiGene™ kit (BIOFACT, Korea, cat.RP101). Complementary DNA (cDNA) from the total RNA (1 ug) was synthesized with a cDNA synthesis kit (Bio-rad, USA, cat.1708891). RT-qPCR was performed using specific primers and SYBR Green (Bio-Rad, cat. 1725271). The primer sequences used were in Table S1. Calculation of a change in the mRNA expression level after correcting for actin was performed as a fold-change ratio, compared to the control.

Table S1. Primer sequences used in qRT-PCR analysis

Gene	Primer	Oligonucleotide Sequence (5'-3')
mActin	F	TGGAATCCTGTGGCATCCAT
	R	TAAAACGCAGCTCGTAACAG
mRANTES	F	GCTCCAATCTTGCAGTCGTGTT
	R	ATTTCTTGGGTTTCGTGGTCG
mMDC	F	TCTGATGCAGGTCCCTATGG
	R	TTATGGAGTAGCTTCTTCAC
mIL-6	F	CCGAAGTCATAGCCACAC
	R	TCCAGTTTGGTAGCATCCATC
mTARC	F	AGAATATCTTTCAGGACCCCT
	R	TTGTGTTCGCCTGTAGTGCATA
mFilaggrin	F	TTTCGTGTTTGTCTGCTTGC
	R	GGGACAGCAGGTTCCCAT
hActin	F	GCGGGAAATCGTGCGTGACATT
	R	GATGGAGTTGAAGGTAGTTTCGTG
hMDC	F	AGGACAGAGCATGGCTCGCCTACAGA
	R	TAATGGCAGGGAGGTAGGGCTCCTGA
hIL-6	F	AGAGTAGTGAGGAACAAGCC
	R	TACATTTGCCGAAGAGCCCT
hTARC	F	CTTCTCTGCAGCACATCC
	R	AAGACCTCTCAAGGCTTTG
hFilaggrin	F	TTTCGTGTTTGTCTGCTTGC
	R	CTGGACACTCAGGTTCCCAT

Supplementary method S4. *Immunoblot*

Proteins from the skin tissue and cell lysates were extracted with lysis buffer, according to the manufacturer's protocols (Intron, Republic of Korea, cat.17081). Proteins were separated by gel electrophoresis and then were transferred onto a membrane (Bio-rad, cat. #1704156). The membrane was blocked for 30 min with EzBlock Chemi buffer (ATTO, Japan, cat.AE-1475) and immunoblotting

for detection of individual proteins were allowed to react overnight with the primary antibodies for anti-STAT1 (9175S), anti-p-STAT1 (9167S), anti-STAT3 (4904S), anti-p-STAT3 (9131S), anti-JAK1 (3332S), anti-p-JAK1 (3331S), anti-β-actin (5125S) (Cell signaling, USA), or anti-filaggrin (Enzo, USA, Cat.ENZ-ABS181-0100) at 4ºC. The membrane was then reacted with secondary antibodies (Cell signaling, cat.7074S) for 1h. The detection of band was performed using a ECL detection reagent (Thermo Scientific, USA, cat.34095).

Supplementary Figure S1. *Original western blot of mouse skin*

Figure S1. Original Western blot of mouse skin (1)

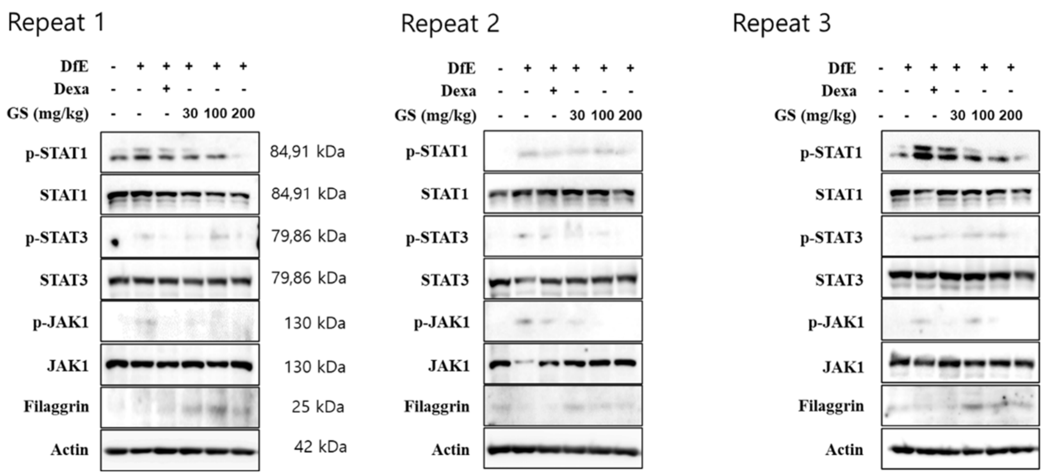

Figure S1. Original Western blot of mouse skin (2)

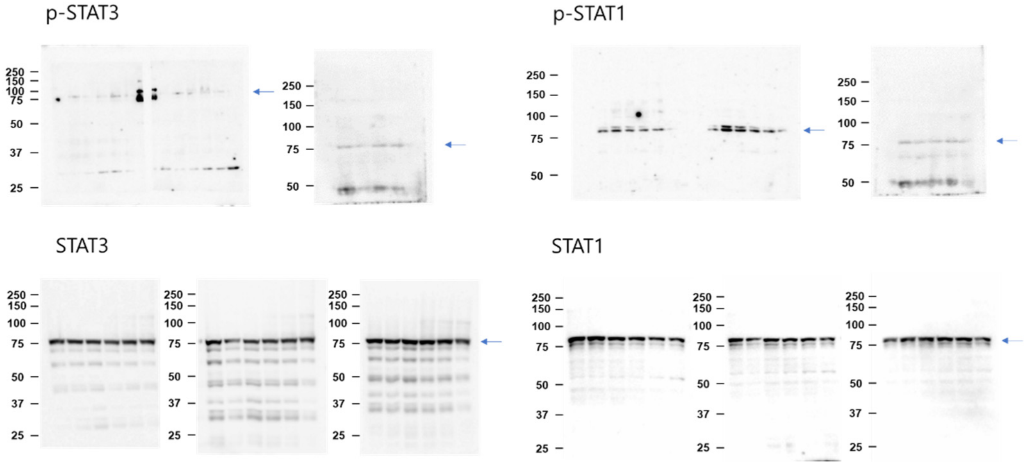

Figure S1. Original Western blot of mouse skin (3)

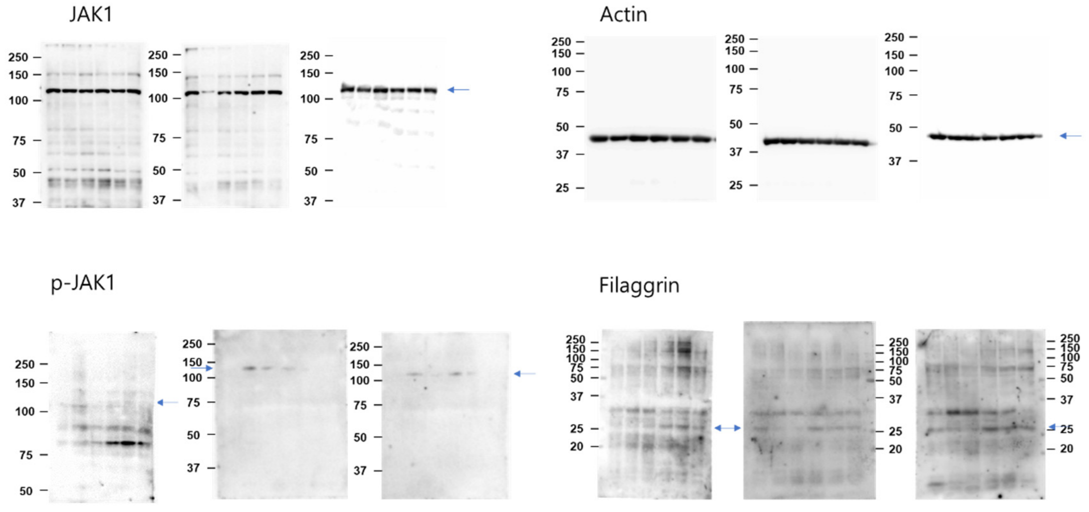

Supplementary Figure S2. *Original western blot of cell lysates*

Figure S2. Original Western blot of cell lysates (1)

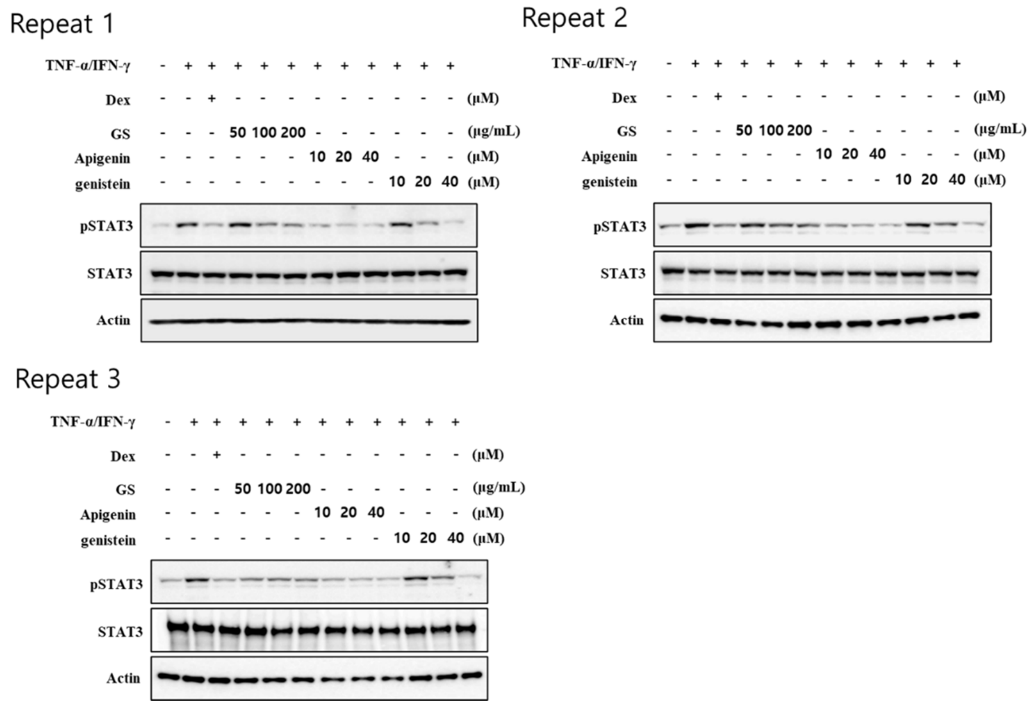

Figure S2. Original Western blot of cell lysates (2)

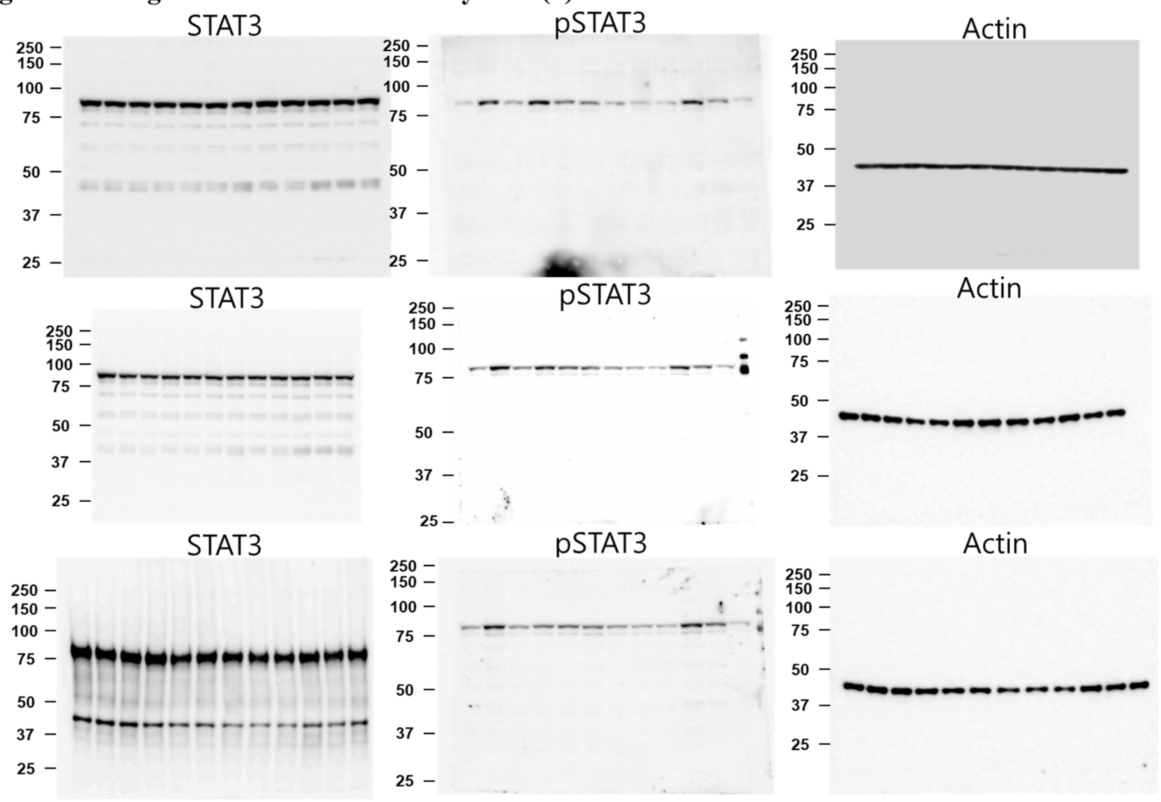

Supplement: Supplementary file 1 [file ijms-26-04560-s001.zip › ijms-3614927-supplementary.pdf]
